# Cancer Stem Cells as a Source of Drug Resistance in Bone Sarcomas

**DOI:** 10.3390/jcm10122621

**Published:** 2021-06-14

**Authors:** Sofía T. Menéndez, Borja Gallego, Dzohara Murillo, Aida Rodríguez, René Rodríguez

**Affiliations:** 1 Instituto de Investigación Sanitaria del Principado de Asturias (ISPA), Hospital Universitario Central de Asturias, Avenida de Roma s/n, 33011 Oviedo, Spain; borjagallego@ispasturias.es (B.G.); dzomc@ispasturias.es (D.M.); aida.rodriguez@finba.es (A.R.); 2 Instituto Universitario de Oncología del Principado de Asturias, Universidad de Oviedo, 33006 Oviedo, Spain; 3 CIBER en Oncología (CIBERONC), 28029 Madrid, Spain

**Keywords:** bone sarcoma, osteosarcoma, cancer stem cells, drug resistance, stemness signaling, tumor microenvironment, metabolism, epigenetics, microRNAs

## Abstract

Bone sarcomas are commonly characterized by a high degree of intra-tumor heterogeneity, which in part is due to the presence of subpopulations of tumor cells presenting stem cell properties. Similar to normal stem cells, these cancer stem cells (CSCs) display a drug resistant phenotype and therefore are responsible for relapses and tumor dissemination. Drug resistance in bone sarcomas could be enhanced/modulated during tumor evolution though the acquisition of (epi)-genetic alterations and the adaptation to changing microenvironments, including drug treatments. Here we summarize findings supporting the involvement of pro-stemness signaling in the development of drug resistance in bone sarcomas. This include the activation of well-known pro-stemness pathways (Wnt/β-Cat, NOTCH or JAT/STAT pathways), changes in the metabolic and autophagic activities, the alteration of epigenetic pathways, the upregulation of specific non-coding RNAs and the crosstalk with different microenvironmental factors. This altered signaling is expected to be translated to the clinic in the form of biomarkers of response and new therapies able to overcome drug resistance.

## 1. Introduction: Cell Heterogeneity and Cancer Stem Cells in Bone Sarcomas

Bone sarcomas comprise a rare group of malignancies, which represent less than 0.2% of cancer diagnoses [1]. Among this group of tumors, osteosarcomas (35% of primary malignant bone tumors), chondrosarcomas (25%) and Ewing sarcomas (16%), each comprising several sub-entities, represent the most common subtypes [1,2]. Osteosarcomas and chondrosarcomas are characterized by a complex and variable genomics, where only few genes, such as TP53, RB, ATXR or PTEN in osteosarcomas [3,4,5] and IDH1/2, COL2A1 or TP53 in chondrosarcoma [6,7], were commonly mutated in a significant number of patients. On the other hand, Ewing sarcomas are genetically stable tumors, characterized by the presence of cytogenetic translocations, involving ETS transcription factors, with EWS/FLI1 being the most common [8]. Despite their relatively low incidence, bone sarcomas represent a medical challenge due to their aggressive behavior and lack of significant improvement in their treatment protocols for decades. Therapeutic options for bone sarcomas have remained largely unaltered since the late 1970s and mainly rely on a surgical resection with adequate margins, combined or not, with pre- and/or post-operative radiotherapy and/or chemotherapy (doxorubicin, ifosfamide, methotrexate, cisplatin, etc.) [9,10]. While, a high proportion of tumors initially respond well to these treatments, more than 30% of patients with localized osteosarcoma and more than 80% with metastatic/relapsed disease still succumb to the disease, owing to the appearance of resistant tumor subclones [10,11].

The majority of osteosarcomas and Ewing sarcomas arise during puberty in areas of actively growing bone areas, like the metaphysis of long bones. Another portion of osteosarcomas and chondrosarcomas develop in adulthood and might be associated with a deregulation of the bone remodeling process [12]. The development of these bone tumors may be linked to an imbalance between the demand of progenitor cells during periods of increased bone formation and remodeling and the limited expansion capacity of normal stem cells [13]. This results in the appearance of pre-malignant stem/progenitor cells, which would alter bone homeostasis and initiate tumor formation after suffering sequential mutations targeting differentiation and proliferation pathways. In this regard, mesenchymal stem/stromal cells (MSCs) represent a subset of adult multipotent stem cells present in the bone marrow and other tissues that constitute a source of progenitors for mesodermic tissues, including bone [14,15]. Experimental evidence suggests that osteosarcomas and chondrosarcomas may arise upon the malignant transformation of MSCs or their derived progenitors along the osteo/chondroblastic lineage [16,17,18,19,20,21,22,23]. There is also evidence regarding the role of MSCs as cell-of-origin for Ewing sarcoma, but a neural origin for these diseases have also been proposed [24,25,26].

Soon after tumor growth initiates, bone sarcomas begin to gain cellular heterogeneity through a process of clonal genetic evolution driven by tumor adaptation to changing microenvironmental conditions, including drug treatments. Furthermore, tumor heterogeneity may also be acquired through a differentiation-driven mechanism guided by a subpopulation of tumor cells that have acquired stem cell-like properties through the action of several genetic and epigenetic influences and microenvironmental signals. [27,28]. Normal adult stem cells, such as MSCs, are long-lived cells that direct continuous tissue remodeling/regeneration processes through the derivation of a panel of specialized, short-lived cells that ultimately perform tissue-specific functions. Similar to normal stem cells, the cancer stem cell (CSC) subpopulations that emerge and evolve during tumor growth are capable of self-renewal and differentiation into less tumorigenic progenies within the tumor. They are also considered to be the only subset of tumor cells able to sustain and (re-)initiate tumor growth [27,29,30]. Both sources of intra-tumor heterogeneity are thought to cooperate to drive tumor growth in a process where different CSC subpopulations coexist and direct the evolution of tumor clones in a highly dynamic process, by which differentiated tumor cells may re-adopt an stemness state. Relevant to the sarcoma development course, the presence of CSCs is linked to drug resistance and tumor recurrence, invasion and metastasis, making them one of the main factors driving the long-term maintenance of the disease [16,31,32,33]. Similar to normal stem cells, CSCs display several properties that may render them resistant to chemotherapeutic drugs and other xenobiotic compounds. This includes: (i) The expression of detoxification mechanisms, such as efflux pumps of the ABC family or aldehyde dehydrogenase (ALDH) enzymes; (ii) a high DNA repair capacity; (iii) the altered expression of apoptotic regulatory factors; and (iv) the adoption of a quiescent state [16,27,29,34,35,36,37]. In addition, the cell plasticity observed in CSC hierarchies may also constitute an important driver of drug resistance. Finally, these properties are also highly influenced by signaling from the surrounding milieu and the physical properties of specific microenvironments [27,29,38,39].

CSCs subpopulations in bone sarcomas have been identified according to the above described stem cell-related properties and/or marker expression [31,33,40,41]. Most common methods used to isolate CSCs in sarcomas include: (i) Culture of floating 3D colonies (sarcospheres), a property associated to the self-renewal ability of stem cells; (ii) the sorting according to the expression of specific surface markers expressed by normal stem cells such as CD133, STRO1, CD117, CD271, ABCG2, etc.; (iii) the isolation of subpopulations with high enzymatic activity of ALDH1; iv) the identification of a “side population” able to exclude fluorescent dyes, a feature associated to the expression and activity of ABC pumps; or (v) the tracking of subpopulations that express stem cell-associated genes. Isolated CSC subpopulations should be able to regenerate non-stem cells, present in the initial culture, display increased levels of pluripotency markers (e.g., SOX2, OCT3/4) and drug efflux transporters (e.g., ABCG2), and demonstrate enhanced ability to initiate tumor growth in vivo. In any case, it should be noted that CSCs in bones sarcomas are heterogeneous and the different methods may involve selecting different CSCs subpopulations within the tumor mass. Interestingly, whichever isolation method was chosen, CSCs subpopulations usually proved to be more resistant to drugs employed in the clinical treatment of bone cancer, such as doxorubicin, methotrexate or cisplatin [42,43,44,45,46,47,48,49,50,51,52,53,54,55,56,57,58] (Table 1). Moreover, resistant bone sarcoma models, generated by serial exposure to chemotherapeutic drugs, show exacerbated stemness qualities when compared with their non-resistant counterparts [36,59,60,61,62,63,64,65,66] (Table 2). Besides, it has been established a correlation between expression of stemness markers and a worse response to chemotherapy in osteosarcoma patients [62].

Altogether, these data provide evidence for a link between stemness and drug resistance in bone sarcomas. Below, we will review different pro-stemness mechanisms used by CSCs to develop drug resistance in bone sarcomas.

## 2. Mechanisms Involved in Cancer Stem Cell-Mediated Drug-Resistance

### 2.1. Stemness-Related Signalling Pathways

**Figure 1:**
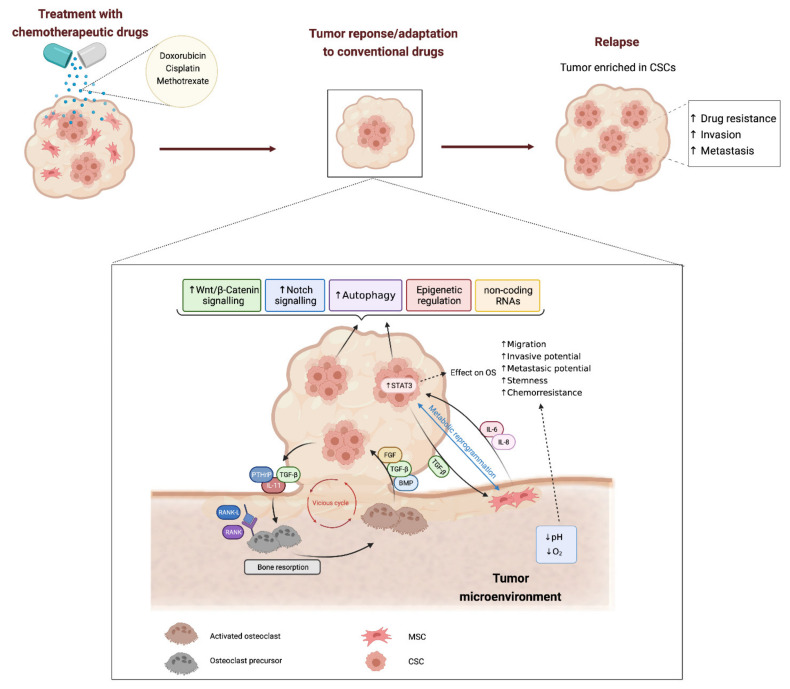
Mechanisms involved in Cancer Stem Cell-mediated drug-resistance in bone sarcomas. Tumor response to chemotherapeutic drugs is frequently characterized by the activation of stemness-related pathways such as WNT/β-catenin and Notch signalling. Autophagy, epigenetic regulation and the expression of certain non-coding RNAs may also modulate CSC response to anti-cancer therapies. Within the bone microenvironment, the crosstalk between sarcoma cells and the cell types that regulate bone homeostasis may initiate a vicious circle resulting in a dysregulated bone lysis and the release of bone matrix growth factors (BMP, TGF-β or FGF) that promotes tumor growth and stemness. In addition, the crosstalk signalling between MSCs and tumor cells may also result in increased drug resistance and more aggressive phenotypes (higher invasive and metastatic potential) through the activation of pro-stemness factors, such as STAT3, or the metabolic reprogramming in sarcoma cells. Finally, hypoxic and/or acidic microenvironments may also contribute to CSC-mediated drug resistance in bone sarcomas.

Several studies have contributed to establish a link between therapy resistance and abnormal activation of growth and/or survival signaling pathways in CSCs [67] (Figure 1). Among them, the Wnt/β-Catenin pathway is the most studied in osteosarcoma regarding their role in drug resistance. Conventional chemotherapeutic drugs such as doxorubicin, cisplatin and methotrexate, effectively induce the expression of stem cells markers (i.e., SOX2, OCT4, KLF4 or Nanog) and multidrug resistance-related transporters (i.e., ABCG2 or ABCB1) in osteosarcoma cells through the activation of the Wnt/β-Cat pathway [62]. Interestingly, disruption of this pathway with the tankyrase inhibitor IWR-1 reverses these effects by reducing the levels of AXIN2, a negative regulator of the Wnt signaling, and results in impaired CSCs proliferation and viability. Moreover, this treatment was able to re-sensitize osteosarcoma CSCs to doxorubicin, both in vivo and in vitro [68]. Similarly, the β-catenin/transducin β-like protein 1 (TBL1) inhibitor tegavivint reduced the expression of ALDH1 and impaired primary tumor growth, as well as distal metastatic development in two PDX mice models derived from doxorubicin-resistant tumors. Consistently, this treatment also restored the sensitivity of these cells to doxorubicin and the combination of both drugs resulted in increased survival of mice models [69]. Another study showed that knockdown of the three prime repair exonuclease 1 (TREX1) enhanced stemmness properties and resistance to cisplatin and doxorubicin in osteosarcoma cells through the E2F4-mediated activation of the Wnt/β-catenin pathway [70]. Other factors have also been involved in the regulation of the Wnt/β-catenin-mediated induction of stemness in rare subtypes of bone sarcomas. This is the case of the melatonin specific membrane receptor 1B (MTNR1B) in recurrent chordoma where this receptor has been found downregulated and inversely correlated to the levels of OCT4. Moreover, its levels were lower in spheres than in the corresponding adherent cells [71]. Interestingly, the activation of MTNR1B could sensitize cells to cisplatin as it mediates melatonin-induced inhibition of the Wnt/β-Catenin pathway by preventing β-Cat phosphorylation by SRC. In line with this finding, the combination of cisplatin with melatonin or dasatinib (a SRC inhibitor) resulted in an improved response to chemotherapy in mice xenografts [71].

Notch signaling also plays relevant roles in stem cells-mediated resistance in osteosarcoma. This pathway has been found activated in resistant osteosarcomas, where it was positively correlated to stem cell-like properties. The expression of Notch1 intracellular domain (NICD1) and several Notch targets like Hes1, Hes5 and HeyL, were found to gradually increase in normal adjacent tissues, chemosensitive tumors and chemoresistant tumors. Besides, the mRNA levels of OCT4 mimicked this gain pattern too, suggesting a link between resistance and stemness in osteosarcoma [72]. Consequently, two studies found that different γ-secretase inhibitors (GSI), not only decreased the CSC-like phenotype but also restored chemosensitivity in cisplatin-resistant cell lines [66,72]. As a clue to the mechanism behind this re-sensitization, blockade of NOTCH signaling with GSI was found to be associated to the inhibition of AKT and ERK signaling [72]. A subsequent study confirmed that Notch overexpression in osteosarcoma cells is linked to chemoresistance along with increased tumorigenicity, invasion and stemness. The authors also demonstrated that this role of Notch signaling in osteosarcoma was mediated by Ephrin B1 [73].

Other studies have shown the involvement of the JAK/STAT pathway in osteosarcoma resistance. It has been found that Interleukin 6 (IL-6) promotes epithelial-to-mesenchymal transition (EMT), stemness and chemoresistance in osteosarcoma cells through the hyperactivation of STAT3 through a mechanism mediated by osteopontin (OPN) [74]. Relevantly, the treatment of osteosarcoma CSCs with cinobufagin, a steroid lactone used in Chinese medicine, was able to reduce their levels of IL-6, p-STAT3 and OPN, resulting in an attenuated stem-like phenotype and decreased tumor growth [75]. Osteosarcoma cells might also gain stemness properties and tumorigenic potential upon drug treatment through the activation of a VEGF/VEGFR1/ERK autocrine signaling [64]. This work shows that the block of this signaling through the depletion of VEGFR1 or the inhibition of ERK signaling led to the reduction of CSC-associated properties and an enhanced response to cisplatin in vivo [64].

Several members of the Krüppel-like family of pluripotency factors also plays relevant roles in regulating the CSC phenotype and drug resistance in osteosarcoma. Thus, KLF4 expression was associated to the development of drug-induced stemness phenotypes in osteosarcoma cells via a mechanism that seems to be mediated by the activation p38 MAPK signaling [76] and can be inhibited by statins [61]. Similar findings have been reported for KLF8, which was described to control cancer stem cell-like features through a signaling axis involving the regulation of SOX2 expression by miR-429 [77]. In addition, the oncogenic gain-of-function of the tumor suppressor TP53 has been associated with an increase in CSC subpopulations in colon cancer cells treated with doxorubicin [78]. In bone sarcomas, the presence of TP53 gain-of-function mutants have been found to promote a stemness phenotype in a drug-resistant osteosarcoma model [79]. In this line, the reactivation of a functional TP53 pathway underlay the antitumor effect observed after triggering CD99 signaling in Ewing sarcoma. This mechanism, which appears to be specific for tumor cells, also resulted in a significantly increased response to doxorubicin [80]. Finally, the role of efflux pumps of the ABC family in mediating drug resistance in osteosarcoma CSCs has recently been addressed. This study proposed that the acquisition of a multidrug resistance in osteosarcoma is a multi-step process where the expression of different components of the ABC family of transporters are mediating distinct resistant phenotypes [36]. Furthermore, it has been suggested that the high apoptotic threshold of osteosarcoma stem cells to doxorubicin treatment is mainly dependent on the drug concentration reached inside tumor cells which is governed by efflux transporters activity. Therefore, the inhibition of the expression of ABC pumps may result in an enhanced uptake of doxorubicin accompanied by the up-regulation of pro-apoptotic protein BAK, the suppression of anti-apoptotic BCL-2 and increased commitment of CSCs towards apoptosis [81].

### 2.2. Regulation of Metabolism

CSCs have often been reported as quiescent, slow-growing cells that present a low metabolic rate when compared with the highly proliferative cells shaping the bulk of the tumor [37,82]. Through quiescent states, CSCs could survive treatments with chemotherapeutic drugs, aimed to target rapidly dividing cells. Therefore, quiescence provides an advantage to CSCs, as well as an opportunity for cancer to regrowth after therapy. Sarcospheres, derived from MNNG/HOS cells, had low metabolic activity following irradiation, as determined by fluoro-deoxyglucose (FDG) uptake, which was also accompanied by low production of reactive oxygen species (ROS) [49]. These results not only suggest that sphere-forming cells could be kept in a quiescent state, but also that they might develop improved intrinsic antioxidant capacity, which could offer them resistance to conventional therapies that target proliferating cells. In line with this reasoning, the inhibition of metabolic activity with 2-deoxy-D-glucose (2DG, a glucose competitive inhibitor) or metformin (an oral biguanide medicine widely used for type 2 diabetes treatment) can increase the sensitivity of bone sarcoma stem cells to conventional chemoterapeutic drugs [83,84]. Metformin treatment of stem cells isolated from HOS, Saos-2 or MG-63 osteosarcoma cell lines lead to a significant reduction in the IC50 value for cisplatin, doxorubicin and 5-fluorouracil. This sensitization was caused by a weakening of the glucose metabolism that was mediated by glycolytic enzyme pyruvate kinase M2 [84]. Interestingly, metformin could also attenuate the stemnes phenotype in osteosarcoma cells. Therefore, the treatment of osteosarcoma cells with this drug resulted in a reduced formation of self-renewing sarcospheres, a decreased expression of pluripotency markers (Nanog, OCT3/4) and the induction of cell death in CSC subpopulations [85,86]. These effects were mediated by the activation of AMPK and the subsequent inhibition of mTOR signaling and autophagy dysregulation [85,86]. The same effect was detected in Ewing sarcoma cells, since the suppression of the stemness-related phenotype was evident after treatment with 2DG, metformin or a combination of both. In this case, the proportion of cells displaying high ALDH activity, the number of sarcospheres or the mRNA levels of OCT3/4, SOX2 and Nanog dropped significantly with the treatment. Importantly, 2DG was also able to raise the efficacy of doxorubicin and the PARP inhibitor talazoparib [83].

### 2.3. Autophagy

Moreover, autophagy, which related to the regulation of metabolic homeostasis, is a self-degradative process, crucial for balancing sources of energy at critical times, both in the development and in response to nutrient stress. It has been demonstrated that autophagy participates in the homeostasis of osteosarcoma CSCs. The number of autophagosomes, as measured by immunofluorescent LC3-II puncta, increased in osteosarcoma CSCs selected either by their improved ability to form sarcospheres [86,87], enhanced ALDH1 activity [88] or augmented expression of the CSC marker CD271 [51,57]. In addition, osteosarcoma CSCs also displayed higher levels of essential genes for autophagy, such as Beclin1, Atg5 or Atg7. As a result, CSCs benefited from increased resistance to unfavorable circumstances, like nutrient scarcity and hypoxia [57,87] or even chemotherapeutic treatment [86,88]. It has been shown that the IC50 for cisplatin or epirubicin in both Saos2- and MNNG/HOS-CD271+ cells decreased after siRNA knockdown of Atg5 or Atg7 [57]. Moreover, autophagy inhibition through treatment with a catechin from green tea (Epigallocatechin gallate or EGCG) successfully re-sensitized CSCs isolated from Saos2 and U2OS cells to doxorubicin. Besides, this treatment also reduced the expression of pluripotency markers and the formation of sarcospheres in osteosarcoma CSCs [88]. Other compounds that are able to dysregulate the authophagic activity, such as the antipsyhcotic drug thioridazine, may also promote cell death through the induction of autosis in osteosarcoma CSCs [87]. In any case, the role of autophagy in osteosarcoma stemness is complex and a role for autophagy as a negative regulator of CSCs under certain circumstances has been also proposed. In this work, metformin-mediated induction of autophagy disturbed the homeostasis of stemness and pluripotency of osteosarcoma CSCs and was suggested to play a role in the anti-tumor mechanisms induced by this drug [86].

### 2.4. Epigenetic Regulation of Stemness in Bone Sarcoma Stem Cells

From DNA methylation to histone modifications, epigenetic events are key to CSCs plasticity as they offer an effective and versatile method for rapidly changing cellular expression programs. In cell lines derived from Ewing sarcoma, endogenous EWS/FLI1 can bound to the promoter region of EZH2 and regulate its expression in a dose-dependent manner [89]. EZH2 is part of the polycomb repressor complex 2, where it contributes to gene silencing through the tri-methylation of lysine 27 on histone 3 (H3K27me3). The blockade of EZH2 expression in Ewing sarcoma cell lines decreased overall H3K27me3 and increased histone H3-acetylation (H3K27ac). Interestingly, this blockade was accompanied by the downregulation of stemness-related genes, such as the nerve growth factor receptor, and the upregulation of genes involved in neuroectodermal and endothelial differentiation like GAP43, EPHB2 or GFAP [89,90]. Moreover, suppression of EZH2 expression in Ewing sarcoma cell lines inhibited contact-independent growth, favored cell differentiation, and reduced tumor growth as well as metastatic dissemination in vivo [82]. These data suggest a role for EZH2-dependent epigenetic regulation in the maintenance of an undifferentiated and stem-like phenotype in Ewing sarcoma, as has been reported in prostate tumors, breast cancer and glioblastoma [91,92]. In addition, higher protein expression of EZH2 was detected in osteosarcoma patients where it significantly correlated with shorter disease free and overall survival. Strikingly, patients with metastasis at the time of diagnosis presented a significant up-regulation of this factor, which was preferentially located at the nucleus [93]. In accordance with this, mRNA levels for EZH2 significantly increased in those osteosarcoma patients who developed metastasis within 5 years after the initial diagnosis. Similar to what was described for Ewing sarcoma, the abrogation of EZH2 expression in osteosarcoma cells was able to decrease cellular growth, migration, invasion and clonogenicity. Furthermore, the depletion of this factor also resulted in a reduction in the levels of the stem cell marker CD44 and Notch3, in addition to the activation of pro-apoptotic pathways [93].

Also in osteosarcoma, the expression of the histone methyltransferase SETD2 has been found to be downregulated in a small cohort of patients when compared with their paired normal tissues [94]. Through the loss and gain of expression experiments, the authors demonstrated that SETD2 acts as a tumor suppressor gene in osteosarcoma cell lines. This factor not only controls tumorigenesis in vivo, but also affects CSCs properties and chemosensitivity by regulating the Wnt/b-catenin pathway [94]. On the other hand, other histone methyltransferase, such as NSD2, have been reported to negatively regulate apoptotic signaling, while enhancing CSC properties and chemoresistance in osteosarcoma cells through a mechanism mediated by H3K36me2 modifications in key apoptotic and pluripotency genes and the activation of ERK and AKT pathways [95]. In addition, nicotinamide N-methyltransferase (NNMT), another methyltransferase previously implicated in different metabolic disorders and cancer development, has also been associated to the acquisition of a stemness state in osteosarcoma [96]. Therefore, the levels of this enzyme were elevated in sphere-forming cells versus control cells and positively correlated with the expression of CSC-associated factors like CD133 or SOX2 [96].

Finally, the leukemia-inhibitory factor (LIF) has been recently reported as an essential factor under the control of super-enhancers that are specific to osteosarcoma [97]. The expression of LIF was significantly higher in osteosarcoma cell lines and tumors and its expression levels were positively correlated to the stem cell core fators SOX2 and Nanog. Moreover, the treatment of osteosarcoma cells with recombinant LIF protein improved sphere-formation, augmented their invasiveness and increased the expression of CSC-related genes, such as CD133, SOX2, Nanog and OCT4. Further investigations have demonstrated that the expression of LIF and downstream pro-stemness effects are regulated by the H3K27me3 demethylase UTX, which is able to join LIF promoter and modulate the super-enhancer signals that control LIF transcription [97]. Therefore, the UTX inhibitor GSK-J4 is able to reduce the endogenous levels of LIF in osteosarcoma cells through in combination of epigenetic signals that affect NOTCH1 signaling, including an increase of H3K27me3 and a decrease in H3K27ac at LIF gene locus. This chemical impairment in LIF expression was accompanied by a reduction in CD133 or CD117 levels and a decrease in the sarcosphere forming potential. Further, spheres treated with GSK-J4 were less tumorigenic than their untreated counterparts when injected in immunocompromised mice. Interestingly, these changes were reverted after the addition of LIF recombinant protein [97]. Overall, the summarized studies highlight the relevant role that epigenetic mechanisms play in the modulation of CSCs properties, including drug resistance in bone sarcomas.

### 2.5. Non-Coding RNAs

**Figure 2:**
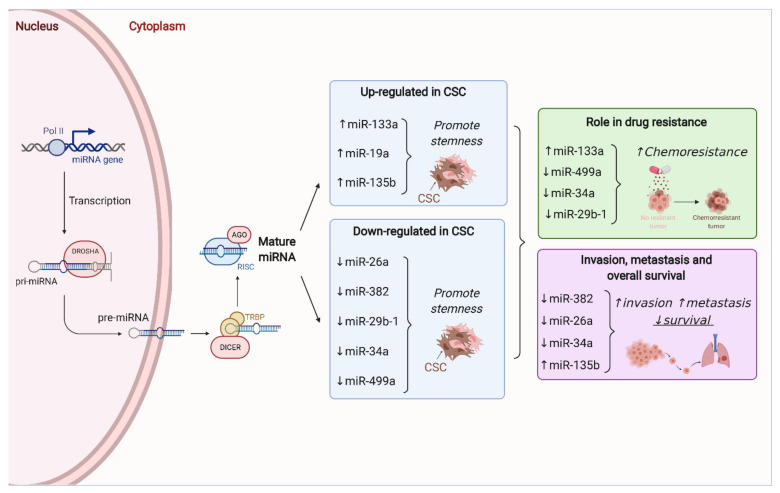
Role miRNAs in stemness and drug resistance in bone sarcomas. MicroRNAs (miRNAs) are regulatory RNA molecules that can simultaneously modulate the expression of a panel of target genes and thus control different cell phenotypes. The process of miRNA transcription and maturation and the enzymes involved in the different steps are shown in the left part of the figure. Right-hand side panels show relevant miRNAs whose upregulation (↑) or downregulation (↓) has been associated with stemness, chemoresistance, metastatic dissemination and/or lower patient survival in bone sarcomas.

MicroRNAs (miRNAs) are small, regulatory RNA molecules that can simultaneously modulate the expression of their respective target genes in a very specific way. Therefore, miRNAs have become key regulators of tumor cell growth, proliferation and survival. Moreover, different studies have demonstrated that miRNAs can modulate the sensitivity of CSCs to anti-cancer therapies [98]. In bone sarcomas, an increasing collection of studies are contributing to define the key role that miRNAs play in the regulation of CSC subpopulations (Figure 2).

The analysis of the miRNA profiles of CSC (CD133high) and non-CSC (CD133low) subpopulations derived from osteosarcoma SaOS2 cells, identified 20 miRNAs that were upregulated in CD133high cells [45]. Among them, miR-133a was also found to be upregulated in the CD133high fraction of osteosarcoma biopsies and was significantly correlated with poor prognosis. Strikingly, miR-133a levels in another osteosarcoma cell line (143B) augmented after treatment with doxorubicin or cisplatin, thereby suggesting the role of this miRNA in the chemoresistance phenotype of osteosarcoma cells. To further test this possibility, the authors treated with cisplatin orthotopic xenografts generated from 143B cells silenced for miR-133a. The combination of chemotherapy with the abrogation of miR-133a expression effectively inhibited the formation of lung metastasis and prolonged mice survival. Moreover, the mRNA of four direct targets of miR-133a (SGM2, UBA2, SNX30 and ANXA2) showed an inverse response to the levels of this miRNA in osteosarcoma cells and were correlated with poor prognosis in patients [45].

Adopting a similar approach, another study identified miR-499a as a key factor in the regulation of the resistance to EGFR inhibitors, such as erlotinib in osteosarcoma CSCs [99]. The authors showed that CD166 marks a subpopulation of osteosarcoma cells with stem-like properties that were also resistant to erlotinib thanks to a TGFβ-induced EMT-associated kinase switch. Subsequent investigation demonstrated that overexpression of miR-499a could revert TGFβ-induced resistance both in vitro and in vivo through the control of SHKBP1 transcription. These results provide a rationale for using the SHKBP1/miR-499a ratio as a predictive biomaker of response to erlotinib in osteosarcoma [99].

Besides miR-133a and miR-499a, other miRNAs have been related to the maintenance of the CSC phenotype in osteosarcoma. Upon upregulation, the levels of miR-29b-1 [100], miR-382 [101], miR-26a [102] and miR-34a [103], impair CSCs growth and self-renewal capability as well as induce a decrease in the expression of stem cell markers like SOX2, CD133, ALDH1, Nanog or OCT3/4. Interestingly, high levels of miR-29b-1, miR-382 and miR-26a were also associated with increased sensitivity to doxorubicin, and in the case of miR-29b-1, also to cisplatin and etoposide [100,101,102]. Noteworthy, overexpression of all miRNAs but miR-29b-1, seem to have an impact over CSCs migration and invasion abilities. Upregulation of miR-382 decreased invasion both in vivo and in vitro by reducing the expression of EMT markers like vimentin and fibronectin. Interestingly, the diminished expression of this miRNA is frequently observed in osteosarcoma patients that suffer from metastatic relapse, and its levels correlate directly to metastasis-free survival and inversely to recurrence rate [101]. Elevated levels of miR-26a are also associated with better survival and reduced risk of metastasis in osteosarcoma patients, which is in agreement with the decrease in tumor latency observed in vivo for osteosarcoma cells that overexpress miR-26a [102]. Although there are no clinical data for miR-34 in osteosarcoma, it has been reported that its overexpression reduces the tumorigenic potential of osteosarcoma cells while favoring the expression of osteogenic markers. Similarly, miR-34 has been suggested to work as a tumor-suppressor factor in Ewing sarcoma patients [104]. Its expression was typically lower in patient samples with respect to the healthy controls, and even lower in cases with metastatic disease. Moreover, miR-34 was significantly correlated with the risk of recurrence as those patients displaying high levels of the miRNA presented increased disease-free survival. Interestingly, the authors also found that response to chemotherapy was slightly better in tumors bearing high levels of miR-34 [104].

On the other hand, the upregulation of miR-19a and miR-135b causes the opposite effect in osteosarcoma CSCs. CD133+ cells displayed higher levels of miR-19a than their negative counterparts. In addition, knockdown of this miRNA significantly decreased the proportion of CD133+ cells and regulate cell proliferation, migration and viability in osteosarcoma CSCs by modulating the PTEN/PI3K/Akt pathway [105]. A similar behavior was observed for miR-135b. Its overexpression not only favored sarcosphere growth and cell invasion, but also augmented the expression of CD133, ALDH1, Nanog and OCT4 [106]. In mice, suppression of miR-135b effectively abrogated CSC-induced tumorigenesis, lung metastasis formation and relapse after cisplatin treatment. Moreover, in clinical samples, high levels of miR-135b were associated with poorer disease-free and overall survival of osteosarcoma patients. It is worth mentioning that the authors also found that the effects seen upon miR-135b upregulation could be reverted by miR-200, which negatively regulated Notch signaling in osteosarcoma cells [106].

Besides miRNAs, other types of non-coding RNAs, such as long non-coding RNAs (LncRNAs), have been involved in the regulation of stemness and drug-resistance in bone sarcomas. DANCR and MALAT1 are two lncRNAs that have been found to promote stemness in osteosarcoma. The levels of both lncRNAs are positively correlated with the mRNA expression of CD133, SOX2 or CD90, and their upregulation in osteosarcoma cells led to an increase in the formation of sarcospheres [107,108]. Interestingly, the effects of both lncRNAs are exerted through activation of the Akt signaling due to the competitive binding of the lncRNA to a regulatory miRNA. Although the effectors are different for each of them. While the binding of DANCR to miR-33a-5p results in higher levels of AXL, a member of the Tyro3-Axl-Mer (TAM) receptor tyrosine kinase subfamily [108], MALAT1 augmented the expression of RET proto-oncogen by competitively binding miR-129-5p [107]. The expression of both lncRNAs was higher in osteosarcomas than in the tissue adjacent to the tumor. Moreover, their expression was correlated with reduced disease-free and overall survival [107,108]. A third lncRNA, the SOX2 Overlapping Transcript variant 7 (SOX2OTv7) has been linked to stemness in osteosarcoma [88]. The overexpression of this lncRNA resulted in enhanced sphere formation in U2OS and SaOs2 cells. Moreover, SOX2OTv7 levels increased after doxorubicin treatment and contributed to trigger a doxorubicin-induced pro-survival autophagic activity. Importantly, EGCG may reduce the stemness properties and improve the anti-proliferative effects of doxorubicin in osteosarcoma cell lines by decreasing SOX2OTvt7-associated signaling [88]. A role for other lncRNAs has been reported, such as MSC-AS1 and TTN-AS1 in osteosarcoma progression and drug resistance [109,110]. Although, the involvement of osteosarcoma CSCs in the drug resistance phenotype mediated by these lncRNAs has not been addressed, the silencing of lncRNA MSC-AS1 resulted in the inactivation of pathways with reported roles in stemness such as PI3K/AKT signaling [110].

Finally, circular RNAs (circRNA) are covalently closed RNA molecules that have also been recently reported to have roles in cancer stemness and drug resistance [111]. In osteosarcoma, several circRNAs has been found upregulated in chemoresistant models and related to poor prognosis, proliferation, invasion and drug resistance [112,113,114,115]. Among them, circUBAP2 were reported to enhance resistance to cisplatin in osteosarcoma through a mechanism involving the activation of pro-stemness pathways, such as WNT signaling [116].

## 3. Influence of Tumor Microenvironment in CSCs and Drug Resistance in Osteosarcoma

Bone sarcomas emerge in a rich environment where tumor cells are closely interacting with with local microenvironmental cell types, such as MSCs, cancer-associated fibroblasts (CAFs), osteoblasts, osteocytes, osteoclasts, chondrocytes, or immune infiltrates [38,117]. Specific physical properties of specific bone microenvironment niches, as well as, supportive signaling generated by the crosstalk between tumor cells and the surrounding milieu, are known to play key roles in the gain of heterogeneity and stemness and the acquisition of drug resistant phenotypes [17,38,118,119,120,121] (Figure 1). A paradigmatic example of the interaction of tumor cells with the microenvironment is the ‘‘vicious cycle’’ initiated by tumor cells with osteolytic potential. These cells are able to produce paracrine factors (like PTHrP, TGF-β or IL11) that stimulate bone resorption through a RANKL–RANK-mediated activation of osteoclasts. The subsequent bone lysis results in a dysregulated release of growth factors from the bone matrix (BMP, TGF-β or FGF), which in turn, may enhance tumor growth and promote stemness in cancer cells [38,118,120]. Also, a low oxygen environment may constitute a supportive niche for bone sarcoma CSCs and alter the response to anti-tumor drugs. Thus, osteosarcoma cell lines cultured under hypoxic conditions may develop resistance to different chemotherapeutic agents through mechanisms dependent or not on the activation of the hypoxia-inducible factor 1α (HIF1α) [122,123]. Again, WNT/b-catenin signaling seems to be key in the mediation of hypoxia-induced chemoresistance in osteosarcoma cells [124].

Within the osteosarcoma microenvironment, MSCs is the cell type with more precisely described interactions with osteosarcoma stem cell subpopulations. MSCs may secret a wide array of growth factors that are able to regulate the proliferation and/or the differentiation of themselves and other components of the bone microenvironment [118,125,126,127]. Conditioned medium (CM) from MSC cultures or extracellular vesicles (EVs) extracted from this CM [128] have been shown to protect osteosarcoma cell lines against death caused by nutrient deprivation [129,130] or drug-induced apoptosis [131,132]. In addition, it was demonstrated that MSCs activate the proliferation of osteosarcoma cells in vitro as well as accelerate local growth in vivo [131,133]. Furthermore, CM and EVs from MSCs may enhance the migration [129,134] and invasive potential [134,135] of osteosarcoma cells. Accordingly, MSCs may interact with osteosarcoma CSCs to increase the levels of adhesion molecules, such as the intercellular adhesion molecule 1 (ICAM-1), that are responsible for tumor extravasation [136]. These findings might explain the higher metastatic potential observed for osteosarcoma cells exposed to or co-injected with MSCs [134,137,138]. Interestingly, MSCs can also exacerbate stemness in osteosarcoma cell lines. Therefore, the exposure of osteosarcoma CSCs to MSCs result in an increased expression of stem factors (i.e., Nanog, OCT4 or SOX2) and enhanced sphere formation potential [136,139].

The mechanisms underlying the role of MSCs in promoting aggressiveness, recurrence and metastatic potential in bone sarcoma cells are in part dependent on the release of bioactive EVs containing anti-apoptotic proteins, bioactive lipids, mi-RNAs and lncRNAs [129,130]. Two miRNAs that have been related to cell survival and proliferation, miR-21 and miR-34a, were detected in EVs derived from serum-deprived MSCs [130]. Moreover, the lncRNA plasmacytoma variant translocation 1 (PVT1), transported in MSCs-derived EVs, was found to promote proliferation and migration in osteosarcoma cells [140]. The authors demonstrated that EVs containing PVT1 increase the metastatic potential of tumor cells through the upregulation of ETS Transcription Factor ERG [140].

MSCs in bone microenvironment may also be «educated» by bone sarcoma cells through the release of EVs to the microenvironment (Figure 1). In this bidirectional crosstalk, TGF-β-containing tumor-produced EVs are able to induce the production and secretion of pro-tumor factors like IL-6 by MSCs [125,137]. Closing the feedback circuit between MSCs and bone sarcoma cells, several works have identified STAT3 as the main pro-stemness and pro-tumorigenic factor induced by MSC-released IL-6 in osteosarcoma cells [74,132,136,137,141,142,143]. This signaling has also an impact in the development of drug resistance. Thus, it has been shown that STAT3 activation by IL-6 is essential for MSCs-induced chemoresistance. Therefore, blocking of STAT3 signaling by AG490, a JAK2 inhibitor, or the knockdown of IL-6 using siRNA could re-sensitized drug-resistant osteosarcoma cells to doxorubicin and cisplatin [74,132]. Interestingly, the expression of STAT3 was higher in osteosarcoma patients displaying resistant tumors and it was also associated to a poorer outcome [132]. Similarly, serum IL-6 levels were found to be higher in patients presenting metastasis or higher TMN stage [74]. Besides IL6, other MSC-derived factor, such as IL-8, were reported to enhance the pro-tumorigenic properties of bone sarcoma cells. The release of this cytokine from MSCs was shown to promote resistance to cell anoikis and pulmonary metastasis through the activation of the C-X-C chemokine receptor 1 (CXCR1)/AKT pathway [138]. Finally, MSCs may also be re-educated by osteosarcoma cells through a mutual metabolic reprogramming process, which may result in an increased acidification of tumor microenviroment [144]. Notably, tumor acidosis is also an important pro-tumor factor, which may promote survival, chemoresistance and stemness of osteosarcoma cells by acting directly in tumor cells or by activating the NFκB/IL6 axis in MSCs [145,146].

Altogether, these findings highlight the relevance of the bone microenvironment in the development of osteosarcomas and the modulation of their response to anti-tumor treatments. Therefore, different therapies aimed to counteract the pro-tumorigenic signaling between tumor cells and the surrounding microenvironment are being explored. These strategies, recently reviewed elsewhere [10,118,127], include the targeting of dysregulated osteoclast activity, hypoxic signaling, angiogenesis, immune system components, including tumor associated macrophages and immune checkpoints. Moreover, blocking the crosstalk between tumor-educated MSCs and tumor cells using IL6- and TGFβ inhibitors may also represent useful therapeutic options to be explored in the future [137].

## 4. Conclusions

There is a consensus about the need to target drug resistant CSCs to reduce the high rate of relapses observed in sarcomas. Here, we recapitulate evidence for intrinsic and drug-induced phenotypic stem cell state transitions that reinforce the suspected link between stemness and drug resistance in bone sarcomas. The inhibition of the pro-resistance mechanisms involved in the acquisition of resistant phenotypes is being investigated as therapies in preventing drug resistance in bone sarcomas.

Further advances are expected to come from “omics” analyses of CSCs, which are still rare in bone sarcoma [65,147,148]. These studies will result in the integration of transcriptomic, epigenomic, proteomic and/or metabolomic data to establish networks of altered signaling in CSCs. In vivo cell tracking of CSC subpopulations and single-cell analyses will also be key for confirming the current knowledge on the topic and ensure new mechanisms driving drug resistance are identified [149,150]. This altered signaling is expected to be translated to the clinic in the form of biomarkers of response and new therapies able to overcome drug resistance in bone sarcomas.

## Figures and Tables

**Table 1:** Reported drug resistance in bone sarcoma CSCs.

Method of CSC Isolation/Characterization	Bone Sarcoma Models	Drug Resistance		Ref.
		Drug	Fold Resistance *	
Sphere formation	MNNG/HOS (OS)	DOX CIS MTX	2.67 2.23 8.33	[49]
	MG63 (OS), HTB166 (EWS)	DOX CIS	N/Q N/Q	[44]
CD133	Human primary, Saos2, MG63, U2OS, HOS, MNNG/HOS, 143B (OS)	DOX CIS MTX	N/Q N/Q N/Q	[45]
	Human primary, STA-ET8.2, TC71, A4573, 5838 and other 5 lines (EWS)	DOX ETO VNC	N/Q N/Q N/Q	[48]
STRO-1/CD177	318-1, K7M2 (OS)	DOX	1.47, 1.73	[42]
CD271	Saos2, U2OS, MNNG/HOS (OS)	CIS	2.16, 1.42, 1.65	[51]
	Saos2, MNNG/HOS (OS)	CIS EPR	N/Q N/Q	[57]
CD24	Human primary, MG63, MNNG/HOS, U2OS, OSC228 (OS)	CIS EPR	N/Q N/Q	[58]
CD49f	UT2, TTC606 (OS)	CIS IDR PTX	6.56, >1.74 1.77, NA 2.10, NA	[55]
Side population	OS1, OS2, OS5 (Human Primary, OS)	DOX CIS MTX	1.33, 1.8, 1.67 1.09, 2.01, 1.07 1.46, 1.43, 0.94	[53]
	Human primary (OS)	DOX CIS MTX	N/Q N/Q N/Q	[50]
	OS65 (OS)	ETO 5-FU CIS PTX GEM OXP	N/Q N/Q N/Q N/Q N/Q N/Q	[52]
	SK-ES-1 (EWS)	DOX CIS	1.64 1.92	[54]
	CADO-ES1 (EWS)	DOX CIS ETO	N/Q N/Q N/Q	[47]
ALDH activity	Human primary (OS/CDS/EWS)	DOX DSF	N/Q N/Q	[46]
	Human primary, TC71, MHH-ES, SK-ES-1, A4573 (EWS)	DOX ETO	N/Q N/Q	[43]
hTERT	Human primary, MG63, MNNG/HOS, 143B (OS)	DOX	N/Q	[56]

* Fold resistance = IC_50_ CSCs/IC_50_ non-CSCs; OS: osteosarcoma, EWS: ewing sarcoma, CDS: chondrosarcoma, DOX: doxorubicin, CIS: cisplatin, MTX: methotrexate ETO: etoposide, VNC: vincristine, EPR: epirubicin, IDR: idarubicin, PTX: paclitaxel, GEM: gemcitabine, OXP: oxaliplatin, DSF: Disulfiram; N/Q: IC_50_ Not quantified.

**Table 2:** Enrichment of CSCs after drug treatment.

Bone Sarcoma Models	Drug Resistance Induction			Characterization of CSC Phenotype	Ref.
	Drug	Method	Fold Resistance *		
MG63 (OS)	3AB	Exposure to 3AB 5 mM for 100 days	N/Q	Sphere formation, pluripot. factors, ABC transporters, CSC markers, MSC features, tumorigenic growth	[59,60]
U2OS, MG63 (OS)	MTX	Exposure to MTX 100 or 300 ng/mL for 5 days	N/Q	Sphere formation, SP, CSC markers, tumorigenic growth	[63]
HOS (OS)	CIS	Exposure to CIS 2 µM for 3 days	N/Q	SP, sphere formation, pluripot. factors, VEGF signaling, tumorigenic growth	[64]
143B, U2OS (OS)	CIS	Exposure to CIS 5 µM for 24 h	1.54, 1.75	Sphere formation, MSC features, pluripot. factors, CSC markers, NOTCH signaling, tumorigenic growth	[66]
HOS, MG63, MHN, MNNG/HOS, OHS, U2OS (OS)	DOX MTX CIS	Exposure to DOX (0.91, 0.31, 0.92, 0.46, 0.52, 0.92 µM), MTX (8, 20, 22, 22, 5, 7 nM) or CIS (3.72, 7.55, 15.87, 3.3, 4.17, 12.48 µM) for 24 h	N/Q N/Q N/Q	ALDH activity, pluripot. factors, ABC transporters, WNT/β-Catenin signaling, tumorigenic growth	[62]
Human primary, U2OS, KHOS/NP (OS)	DOX	Exposure to DOX (50, 100, 100 nM) for 24 h	N/Q	Sphere formation, CSC markers, migration, tumorigenic growth	[61]
HOS, MG63, U2OS (OS)	DOX	Exposure to DOX (14, 28, 28 nM) for 6 months	4.0, 4.0, 6.0	Multidrug resistance, ABC transporters, migration	[36]
MG63 (OS)	DOX	Exposure to increasing doses of DOX (from 2.5 to 1000 ng/mL)	10.53	Multidrug resistance, pluripot. factors, CSC markers, sphere formation, tumorigenic growth	[65]

* Fold resistance = IC_50_ resistant/IC_50_ parental; OS: osteosarcoma, DOX: doxorubicin, CIS: cisplatin, MTX: methotrexate, 3AB: 3-Aminobenzamida, N/Q: IC50 Not quantified, SP: Side population.

## Data Availability

No new data were created or analyzed in this study. Data sharing is not applicable to this article.
